# Preoperative Prevalence and Risk Factors For Calf Muscular Vein Thrombosis in Elderly Patients with Hip Fracture

**DOI:** 10.1111/os.13761

**Published:** 2023-06-13

**Authors:** Sheng Pan, Shen Zhou, Xie‐yi‐dai Ruze, Wang‐yi Jin, Zi‐wen Yan, Da‐lin Peng, Kai‐jin Guo, Yang‐yu‐fan Wang, Xin Zheng

**Affiliations:** ^1^ Department of Orthopaedics The Second Affiliated Hospital of Soochow University Suzhou People's Republic of China; ^2^ Department of Orthopaedics The Affliated Hospital of Xuzhou Medical University Xuzhou People's Republic of China; ^3^ State Key Laboratory of Pharmaceutical Biotechnology, Department of Sports Medicine and Adult Reconstructive Surgery Nanjing Drum Tower Hospital, The Affiliated Hospital of Nanjing University Medical School Nanjing People's Republic of China; ^4^ Department of Orthopaedics, Zhujiang Hospital Southern Medical University Guangzhou People's Republic of China

**Keywords:** Calf Muscular Vein Thrombosis, Elderly, Hip Fracture, Risk Factors

## Abstract

**Objective:**

Increasing evidence has shown that calf muscular vein thrombosis (CMVT) can develop into proximal deep vein thrombosis, even causing pulmonary embolism. However, opinions about the prevalence and risk factors are still controversial. This study aimed to investigate the prevalence and risk factors for CMVT in elderly patients with hip fractures to facilitate their preoperative management.

**Methods:**

We included 419 elderly patients with hip fracture who were treated in the orthopaedic department of our hospital from June 2017 to December 2020. The patients were divided into CMVT and non‐CMVT groups based on color Doppler ultrasound screening of the venous system in the lower extremities. Clinical data, such as age, sex, body mass index, time from injury to admission, and laboratory data were collected. Univariate and multivariate logistic regression analyses were performed to determine independent risk factors for CMVT. A receiver operating characteristic curve was used to analyze the predictive effectiveness of the model. Finally, the clinical utility of the model was analyzed using decision curve analysis and clinical impact curves.

**Results:**

The prevalence of preoperative CMVT was 30.5% (128/419). Independent predictors of preoperative CMVT identified by univariate and multivariate logistic regression analyses were sex, time from injury to admission, American Society of Anesthesiologists (ASA) classification, C‐reactive protein (CRP) level, and D‐dimer level (*p* < 0.05). The area under curve (AUC) was 0.750 (95% CI: 0.699–0.800, *p* < 0.001) and the sensitivity and specificity were 0.698 and 0.711, respectively, which meant the prediction model has a good efficacy in the prediction of CMVT risk. In addition, the fitting degree of the prediction model was also good (Hosmer–Lemeshow χ^2^ = 8.447, *p* > 0.05). The clinical utility of the model was verified using decision curve analysis and clinical impact curves.

**Conclusion:**

Sex, time from injury to admission, ASA classification, CRP level, and D‐dimer levels are independent preoperative predictors of CMVT in elderly patients with hip fractures. Measures should be taken for patients with these risk factors to prevent the occurrence and deterioration of CMVT.

## Graphical abstract

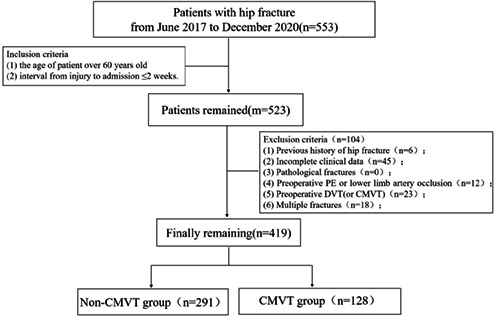

## Introduction

With the aggravation of the aging population, hip fractures have become a major problem in medical care. Venous thromboembolism (VTE), generally including deep venous thrombosis (DVT) of the lower extremities and pulmonary embolism (PE), is one of the most common complications in elderly patients with hip fractures during the perioperative period.^1^ DVT is generally asymptomatic, however, in up to 40% of cases, DVT and PE can occur simultaneously.^2^

Importantly, identifying the risk factors for VTE in the lower extremities before hip fracture surgery could provide valuable information for preventing thrombosis and preoperative optimization, thereby reducing perioperative mortality and related complications. It has been reported^3^, ^4^, ^5^ that factors such as delayed admission or surgery, elevated D‐dimer level, female sex, and lung disease are associated with an increased risk of VTE before hip fracture surgery. Delayed surgery in patients with acute hip trauma is one of the key factors leading to a high incidence of preoperative DVT.^6^

CMVT is commonly considered the starting site for thrombosis of the lower extremities. There is no recommendation of a unified standard for the treatment of CMVT in the latest American College of Chest Physicians guidelines. Statistically, CMVT accounted for approximately 40% of VTE, and about 5%–33%^7^ of PE cases were related to CMVT. CMVT is more common than DVT. Most CMVT cases have no clinical symptoms or signs that are easy to ignore. CMVT is a major risk factor for DVT and pulmonary embolism (PE). Once DVT and PE occur, it can seriously endanger patient safety. Literature has demonstrated that CMVT is independently associated with a higher risk of 30‐day mortality in elderly patients with hip fractures.^8^ Zhuang et al.^9^ evaluated the influencing factors of new‐onset CMVT after hip fractures surgery. However, it is unclear which factors in elderly patients with hip fractures increase the risk of CMVT before surgery.

Accordingly, this study aimed to investigate (i) the prevalence of CMVT in elderly patients with hip fracture and (ii) the potential remediable factors associated with the development of CMVT to help facilitate the perioperative management of patients with hip fractures.

## Patients and Methods

### General Information

This prospective analysis included 553 patients who underwent surgery in the Department of Orthopaedics of our hospital due to hip fracture (including femoral neck fracture and femoral intertrochanteric fracture) from June 2017 to December 2020.

After receiving institutional ethical approval [Clinical Trial Ethics Committee of Affiliated Hospital of Xuzhou Medical University (XYFY2021‐KL127‐01)], all patients provided written informed consent for participation. This study was conducted in accordance with the Declaration of Helsinki of the World Medical Association.

The inclusion criteria were as follows: (1) patients over 60 years of age with unilateral hip fracture (femoral neck fracture or intertrochanteric fracture) and (2) patients with an interval from injury to admission ≤2 weeks.

The exclusion criteria used to select patients were as follows: (1) patients with a previous history of hip fracture; (2) patients with incomplete clinical data; (3) patients with pathological fractures caused by tumors; (4) preoperative PE or lower limb artery occlusion; (5) preoperative DVT; and (6) multiple fractures.

**Fig. 1:**
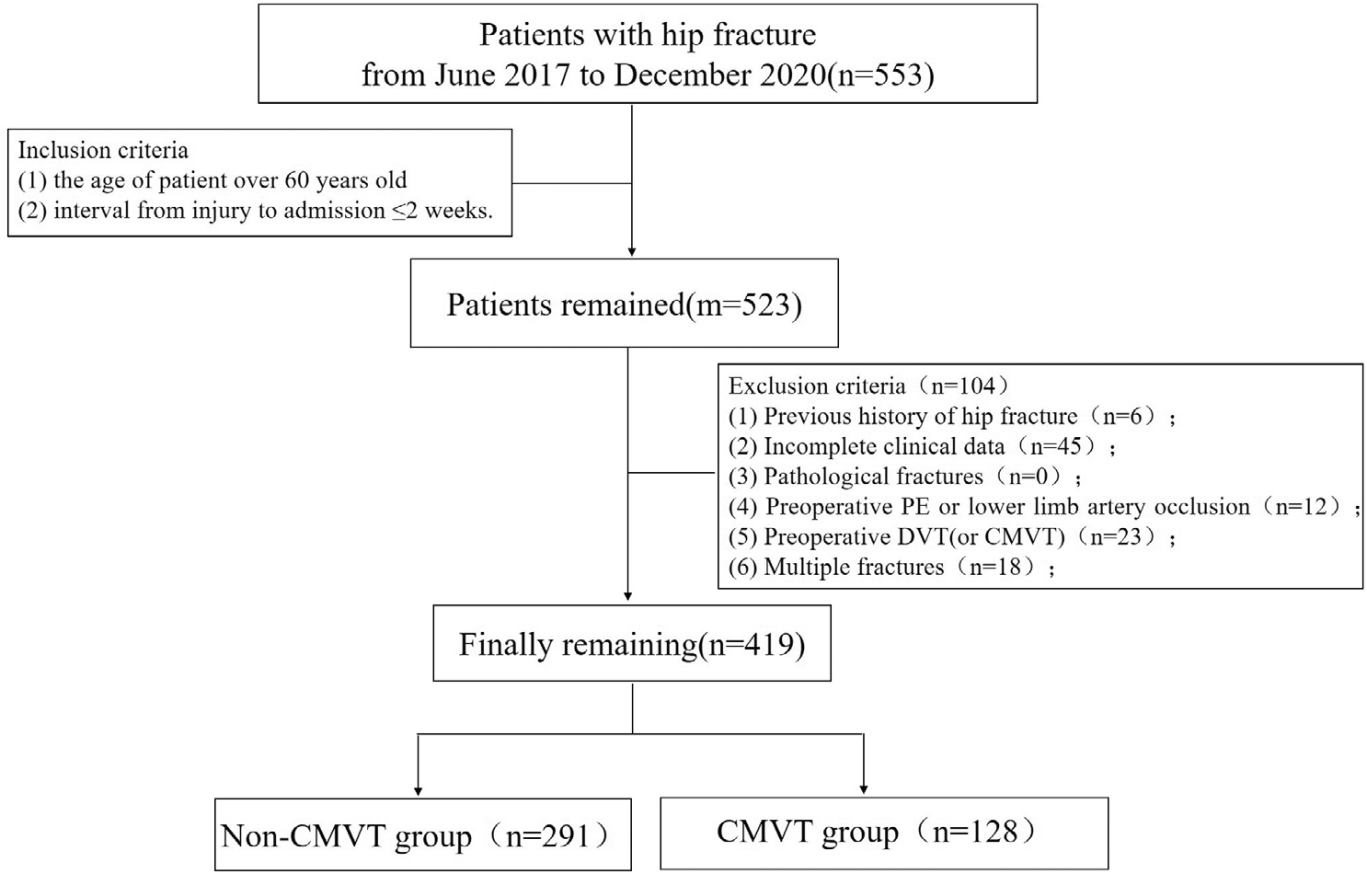
Study flowchart.

All patients underwent chemoprophylaxis and mechanical prophylaxis for DVT at the time of admission and hospitalization. A total of 419 patients were included according to the above inclusion and exclusion criteria. Enrolled patients were further divided into non‐CMVT and CMVT groups according to the results of the color Doppler ultrasound examination after admission (Fig. 1).

### Data Collection

The general data of the enrolled patients included age, sex, fracture type (femoral neck fracture or intertrochanteric fracture), body mass index (BMI), time from injury to admission, underlying diseases (hypertension, diabetes, coronary atherosclerotic heart disease [hereinafter referred to as coronary heart disease], cerebrovascular disease), history of smoking and drinking, and preoperative American Society of Anesthesiologists (ASA) classification.

As suggested by previous research,^10^ patients with ASA classification of grades I–II had a good anesthetic effect and surgical tolerance and recovered smoothly after anesthesia, while patients with grade III–IV showed a gradually increased risk under anesthesia. Therefore, enrolled patients were divided into two groups: grade I–II and grade III–IV group in this study.

Blood samples were also collected and tested in all patients within 1–2 days after admission. Laboratory indices included hemoglobin (HGB), platelets (PLT), C‐reactive protein (CRP), hematocrit (HCT), prothrombin time (PT), international normalized ratio (INR), activated partial thromboplastin time (APTT), thrombin time (TT), fibrinogen (FIB), D‐dimer, albumin (ALB), total cholesterol (TC), and triglycerides (TG). Patients were also divided into two groups: D‐dimer <5 mg/L and D‐dimer ≥5 mg/L, according to relevant clinical experience and previous studies.^11^

### Color Doppler Flow Imaging Examination

All patients underwent color Doppler ultrasound examination of both lower extremities within 1–2 days of admission using a Philips HD15 Color Ultrasound Diagnostic Imaging System with a transducer frequency of 3–10 MHz. During the examination, patients maintained a horizontal position, both lower limbs abduct and extort slightly, and the knee joints maintain natural torsion. From top to bottom, the common femoral vein, superficial femoral vein, popliteal vein, anterior tibial vein, posterior tibial vein, peroneal vein, and venous plexus of the calf muscle were observed by ultrasound probe to detect whether there was dilation and thrombosis. The sites of tenderness were examined carefully for thrombi and the extent of involvement, associated with the observation of the blood flow in the venous lumen.

### Statistical Analysis

Data were analyzed using SPSS 25.0 and R 4.0.4. The normality of the data was tested using the Shapiro–Wilk test. Measurement data that conformed (or approximately conformed) to the normal distribution were expressed as the mean ± standard deviation. The homogeneity of variance tests were simultaneously performed. Intergroup comparisons were performed using two independent sample t‐tests when the variance of the variables of the two groups was homogeneous. Measurement data that did not conform to the normal distribution were expressed as the median (interquartile range) and compared using the Mann–Whitney U test. Furthermore, the counting data were expressed as frequency and percentage, and compared using χ^2^ test, continuity correction, or Fisher's exact test between groups. Potential risk factors for CMVT were identified using univariate analysis. Indicators with a statistically significant difference were then included and analyzed using a multicollinearity test, which were finally subjected to multiple logistic regression analysis to determine the independent risk factors. The prediction efficiency of the model was analyzed using the receiver operating characteristic (ROC) curve, and the calibration curve was drawn by using the “ggstatsplot package” in R 4.0.4. Finally, the decision curve analysis (DCA) and clinical impact curve (CIC) were drawn with the “rmda” package to calculate the net benefit of CMVT at different threshold probabilities before the operation of geriatric hip fracture, so as to confirm the clinical utility of this model. *P* values <0.05 were considered significant.

## Results

### General Data

We included 419 patients, 128 of whom developed CMVT before the operation, with an incidence rate of 30.5%. The 419 cases comprised of 134 men and 285 women, with an average age of 76.0 (68.0, 83.0) years, an average BMI of 24.24 (23.03, 27.76) kg/m^2^, and an average time interval from injury to admission of 24.0 (8.0, 72.0) hours. There were 165 cases of intertrochanteric fractures and 254 of femoral neck fractures. In terms of the medical history of the enrolled patients, there were 174 cases of hypertension, 66 of diabetes, 80 of coronary heart disease, 87 of cerebrovascular disease, 16 of smoking history, and eight cases of drinking history, as well as 84, 276, 57, and two of ASA grades I–IV, respectively.

### Univariate Analysis

There were no statistically significant differences in BMI, fracture type, hypertension, diabetes, cerebrovascular disease, smoking history, drinking history, ASA classification, HGB, PLT, HCT, PT, INR, APTT, FIB, TT, ALB, TG, or TC between the two groups (Table 1 and 2).

**Table 1:** Baseline data and clinical characteristics of the study groups

Variables	Non‐CMVT group (*n* = 291)	CMVT group (*n* = 128)	χ^2^/t/z	*p*
Gender [*n* (%)]			9.147	0.002*
Male	106 (36.4)	28 (21.9)		
Female	185 (63.6)	100 (78.1)		
Age (years)	75.0 (68.0, 82.0)	78.0 (70.0, 85.0)	−2.62	0.009*
BMI (kg/m^2^)	25.45 ± 3.42	25.35 ± 3.55	0.292	0.770
Type of hip fracture [*n* (%)]			2.049	0.152
Intertrochanteric fracture	108 (37.1)	57 (44.5)		
Femoral neck fracture	183 (62.9)	71 (55.5)		
Time from injury to admission (h)	24.0 (7.0, 48.0)	48.0 (19.5, 96.0)	−3.883	<0.001*
hypertension [*n* (%)]	119 (40.9)	55 (43.0)	0.158	0.691
Diabetes [*n* (%)]	40 (13.7)	26 (20.3)	2.889	0.089
Coronary heart disease [*n* (%)]	48 (16.5)	32 (25.0)	4.163	0.041*
Cerebrovascular disease [*n* (%)]	60 (20.6)	27 (21.1)	0.012	0.912
Smoking [*n* (%)]^a^	14 (4.8)	2 (1.6)	1.746	0.186
Drinking [*n* (%)]^a^	8 (2.7)	0 (0.0)	2.270	0.132
ASA classification [*n* (%)]			30.045	<0.001*
I, II	268 (92.1)	92 (71.9)		
III, IV	23 (7.9)	36 (28.1)		

Abbreviations: ASA, American Society of anesthesiologists; BMI, body mass index; CMVT, calf muscular vein thrombosis.

^a^ Indicates that continuity correction has been performed for the χ^2^ test.

* Statistical significance at *p* < 0.05;

**Table 2:** Hematological and biochemical measurements of the study population

Variables	Non‐CMVT group	CMVT group	χ^2^/t/z	*p*
HGB (g/L)	119.0 (107.0, 134.0)	119.0 (103.3, 129.5)	−1.603	0.109
PLT (×10^9^/L)	191.0 (130.0, 237.0)	184.5 (150.3235.0)	−0.158	0.875
CRP (mg/L)	36.00 (14.60, 67.80)	60.25 (21.63, 112.90)	−4.567	<0.001*
HCT (%)	36.5 (32.7, 39.9)	36.1 (32.2, 38.5)	−1.635	0.102
PT (s)	11.3 (10.8, 12.0)	11.3 (10.8, 11.88)	−0.646	0.518
INR	0.99 (0.95, 1.04)	0.98 (0.94, 0.98)	−0.835	0.404
APTT (s)	27.2 (25.4, 29.5)	26.9 (25.9, 29.0)	−0.439	0.660
FIB (g/L)	3.97 (3.24, 4.76)	3.98 (3.36, 4.50)	−0.137	0.891
TT (s)	15.4 (14.9, 16.1)	15.6 (14.9, 16.2)	−1.224	0.221
D‐dimer			7.589	0.006*
<5 mg/L	204 (70.1)	72 (56.3)		
≥5mg/L	87 (29.9)	56 (45.8)		
ALB (g/L)	39.6 (36.5, 42.0)	38.7 (35.6, 40.9)	−1.727	0.084
TC (mmol/L)	4.13 (3.63, 4.80)	3.54 (3.54, 4.61)	−0.408	0.683
TG (mmol/L)	0.97 (0.80, 1.23)	1.04 (0.78, 1.31)	−1.266	0.205

Abbreviations: ALB, albumin; APTT, activated partial thromboplastin time; CRP, C‐reactive protein; FIB, fibrinogen; HCT, hematocrit; HGB hemoglobin; INR, international normalized ratio; PLT, platelet; PT, prothrombin time; TC, total cholesterol; TG, triglycerides; TT, thrombin time.

* Statistical significance at *p* < 0.05.

Statistically significant differences were detected in sex, age, time from injury to admission, coronary heart disease, ASA classification, CRP elevation, and D‐dimer elevation (Table 1 and 2).

### Multicollinearity Analysis

According to the analyses, sex, age, time from injury to admission, coronary heart disease, ASA classification, CRP elevation, and D‐dimer elevation were risk factors for preoperative CMVT in elderly patients with hip fracture. Subsequently, multicollinearity analysis was performed on these risk factors. Considering that the binary logistic module of the SPSS software cannot provide tolerance or variance inflation factors, multiple collinearity between independent variables was tested through linear regression. Generally, the existence of multicollinearity could be determined if the tolerance was <0.1 or the variance inflation factor was >10. As shown in Table 3, there was no multicollinearity among the seven independent variables.

**Table 3:** Results of multicollinearity analysis

Risk factors	Tolerance	Variance inflation factor
Gender	0.993	1.007
Age	0.901	1.110
Time from injury to admission	0.988	1.012
Coronary heart disease	0.829	1.206
ASA classification	0.839	1.192
CRP	0.955	1.047
D‐dimer	0.966	1.035

Abbreviations: ASA, American Society of anesthesiologists; CRP, C‐reactive protein.

### Multivariable Analysis

To screen the risk factors for preoperative CMVT in elderly patients with hip fracture, dichotomous logistic regression analysis was performed with age, sex, interval from fracture to admission, coronary heart disease, ASA classification, CRP, and D‐dimer as independent variables. According to the results of dichotomous logistic regression analysis, the five indicators of sex, time from injury to admission, ASA classification, CRP elevation, and D‐dimer elevation were independent risk factors for preoperative CMVT (Table 4).

**Table 4:** Multivariate logistic regression analysis of independent factors for CMVT

Factors	B	wald	OR (95% CI)	*p*
Female	0.789	8.553	2.202 (1.297–3.736)	0.003*
Age	0.009	0.486	1.009 (0.983–1.036)	0.486
Time from injury to admission	0.007	13.268	1.007 (1.003–1.011)	<0.001*
Coronary heart disease	−0.090	0.076	0.914 (0.484–1.726)	0.782
ASA classification (III、IV)	1.459	21.179	4.031 (2.190–8.446)	<0.001*
CRP	0.010	17.928	1.010 (1.005–1.016)	<0.001*
D‐dimer≥5mg/L	0.640	7.461	1.897 (1.171–3.073)	0.009*

Abbreviations: ASA, American Society of anesthesiologists; CRP, C‐reactive protein.

* Statistical significance at *p* < 0.05 and independent risk factor.

### Construction and Validation of Prediction Model

A new combined predictor model was established with sex, interval from fracture to admission, ASA classification, CRP elevation, and D‐dimer as covariates. The equation was Logit (P) = −3.501 + 0.789 × sex (male = 0, female = 1) + 0.007 × interval from fracture to admission +1.459 × ASA classification (grade I–II = 0, grade III–IV = 1) + 0.010 × CRP + 0.6 × D‐dimer (<5 mg/L = 0, ≥5 mg/L = 1). The omnibus test suggested the overall significance of the model (χ^2^ = 76.053, *p* < 0.001), and the Hosmer–Lemeshow test indicated a high goodness of fit of the model (χ^2^ = 8.447, *p* = 0.391).

**Fig. 2:**
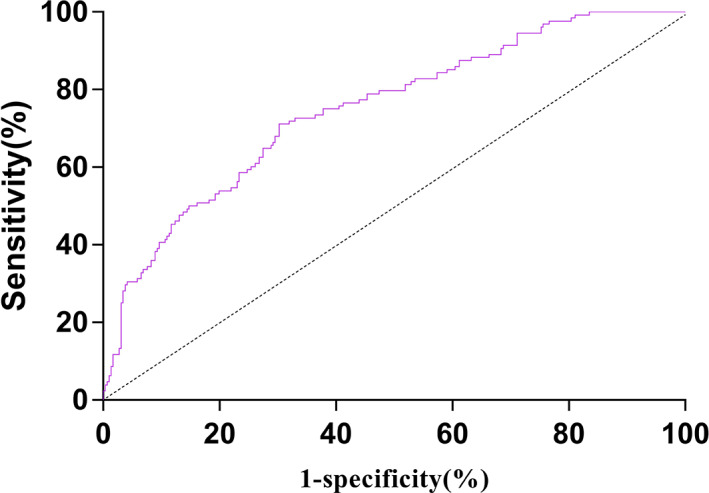
ROC curve of the combined detection factor model.

The area under the curve of the model for diagnosing preoperative occurrence of CMVT was 0.750 (95% CI:0.699–0.800, *p* < 0.001), and the maximum of the Youden index was 0.409. The corresponding sensitivity and specificity were 0.698 and 0.711, respectively, and the cutoff value was 0.282, indicating a good predictive value of this model (Fig. 2).

**Fig. 3:**
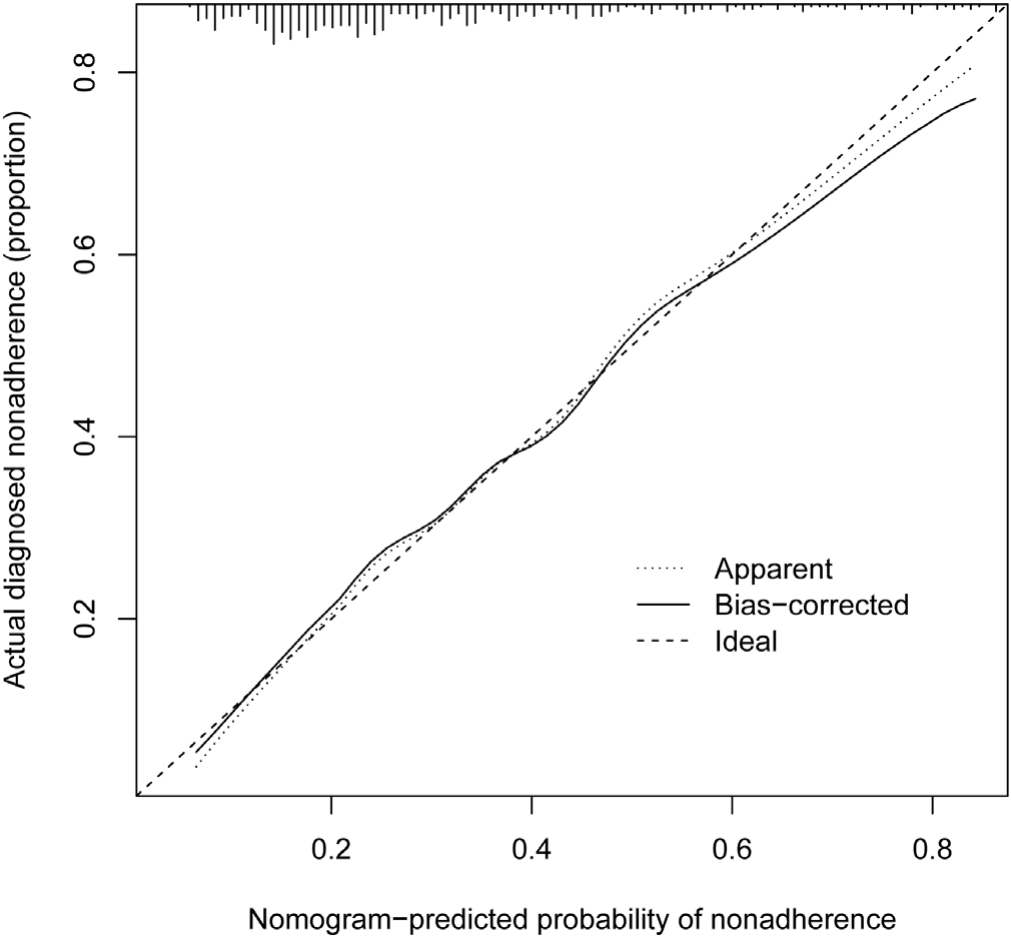
Prediction model calibration chart.

The Bootstrap method was used for internal verification by repeating the sample 1000 times to draw the calibration curve of the prediction model; the X‐axis is the outcome probability predicted by the model, while the Y‐axis is the actual observed value, with 1000 repeated calculations, where the bias‐corrected was the correction curve and the diagonal ideal was the ideal curve, and the prediction ability of the model would be better when the correction curve was closer to the ideal curve. The model exhibited good calibration ability in our study (Fig. 3).

### Clinical Utility of Predictive Models

**Fig. 4:**
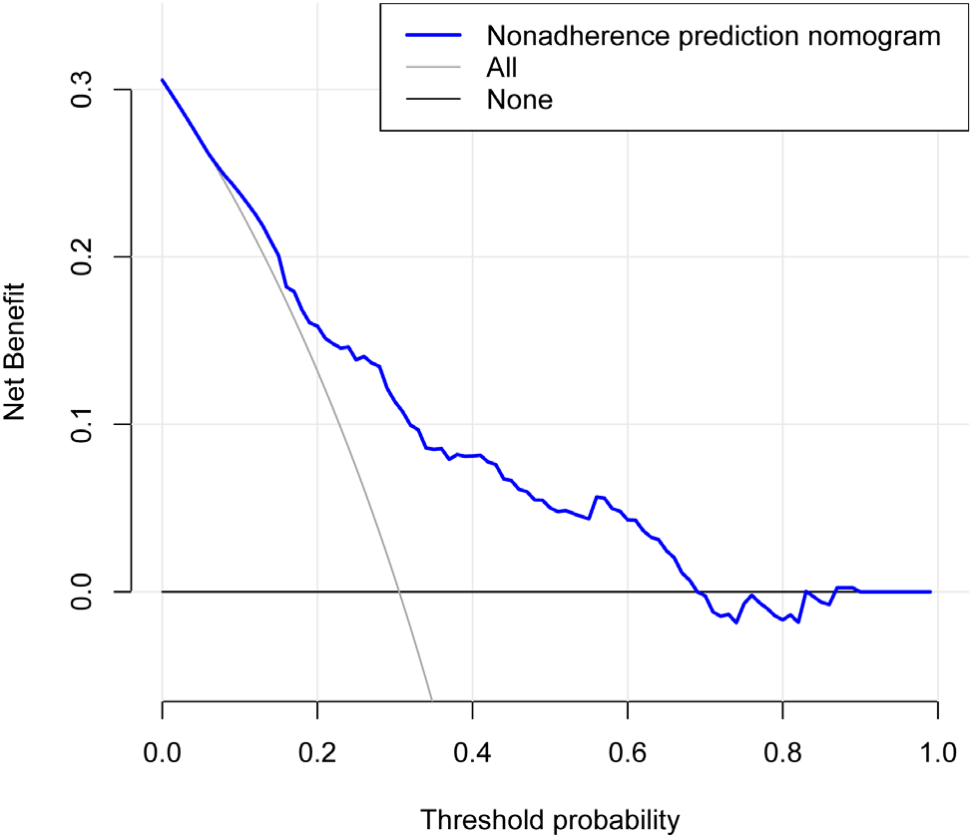
Decision curve analysis.

**Fig. 5:**
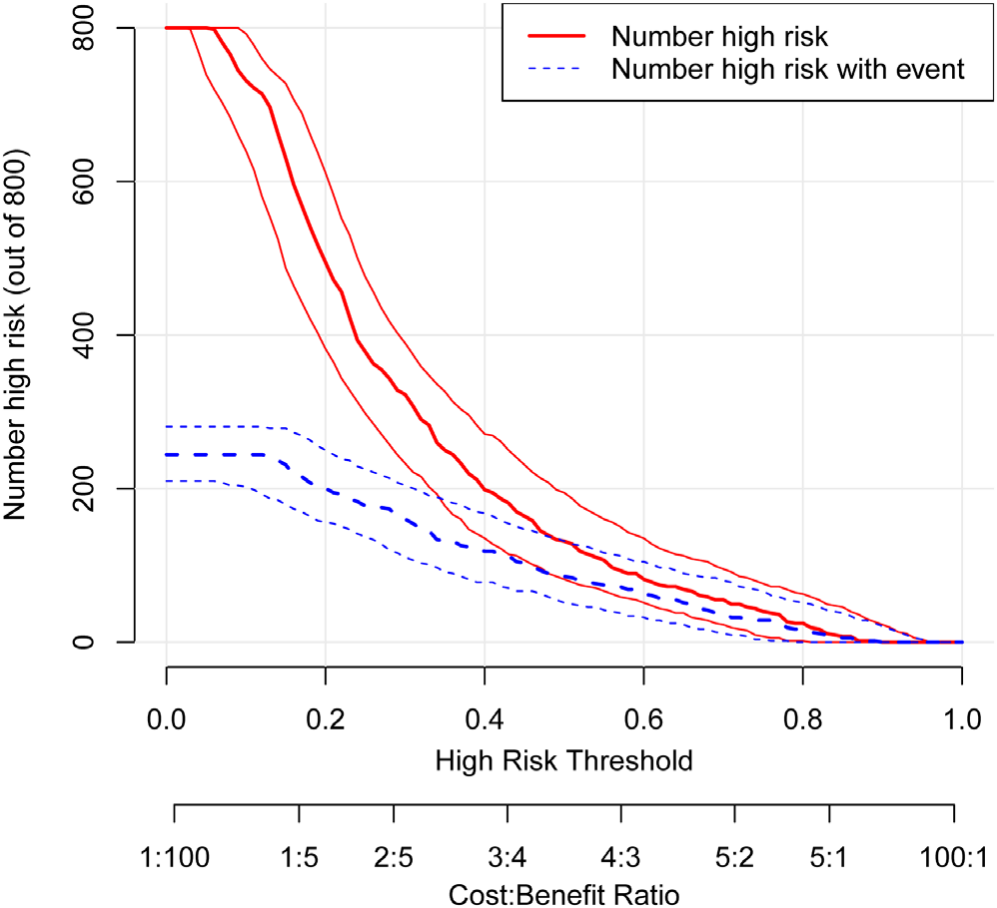
Clinical impact curve.

DCA and CIC were considered novel methods for evaluating the clinical utility of the prediction model, while the blue curve in DCA is the benefit predicted by the model, while the gray curve and the horizontal line curve are the benefit rates of all patients with and without intervention, respectively. The corresponding results revealed the largest clinical net benefit at the threshold of 0.15–0.70 (Fig. 4). Furthermore, the red curve of the CIC is the number of subjects (high‐risk) classified as positive by the model at each threshold probability, and the black curve represents the number of true positives at each threshold probability. The results showed that there were always more patients with expected onset than with actual onset within the threshold probability range (Fig. 5).

## Discussion

The key findings of this study were as follows: (i) we found the prevalence of CMVT was 30.5% in elderly patients with hip fracture; (ii) we identified that sex, time from injury to admission, ASA classification, CRP, and D‐dimer levels were independent predictors of CMVT before surgery in the elderly with hip fracture.

### Prevalence of CMVT

We included 419 elderly patients with hip fracture, and the incidence of CMVT was 30.5%, which was higher than the 9.94%–14.6% reported in the literature.^12^, ^13^, ^14^ This might have been due to the fact that patients diagnosed with DVT or PE after admission did not receive preoperative anticoagulant therapy. Su et al.^12^ did not include cases of femoral neck fractures, and the results of this study showed that the incidence of CMVT was higher in patients with femoral neck fractures than in those with intertrochanteric fractures, although the difference was not significant. Moreover, this study excluded some patients with incomplete data (mostly owing to the lack of color Doppler ultrasound results), which may have affected the results.

### Risk Factors for CMVT

This study found that female sex was a risk factor for CMVT. Whether sex differences affect the incidence of perioperative thrombosis in lower‐limb fractures remains controversial. Most studies have shown that the occurrence of preoperative DVT in the lower limbs is not related to gender.^2^, ^15^ Su et al.^14^ studied the risk factors for CMVT in 312 elderly patients with intertrochanteric fractures and found that female sex was a risk factor for CMVT. Bleker et al.^16^ found that the incidence of lower‐extremity DVT was higher in men than in nonpregnant women. Estrogen can increase platelet viscosity, inhibit the liveness of antithrombin activity, and promote the activity of various clotting factors and thrombin activity.^17^, ^18^ The study included 134 men and 285 women. Therefore, the bias caused by the significant difference in the proportion of males and females is difficult to exclude.

In this study, the time from injury to admission was also a risk factor for CMVT. Su et al.^13^ found that a time from injury to operation of more than 48 hours was a risk factor for CMVT in elderly patients with intertrochanteric fractures (OR = 3.510, *p* = 0.002), which was consistent with our results. The waiting time from injury to surgery can be divided into the time from injury to admission and that from admission to operation. Previous studies^6^ mostly included the waiting time for surgery or the time from admission to surgery as variables in multivariate analyses. We believe that the preoperative color Doppler ultrasound examination of both lower limbs performed before surgery and the surgical delay caused by some reasons after the examination may have biased the results. We believe that if a patient is in good health, surgery should be performed as early as possible after admission. For patients with various underlying diseases, medical institutions should make efforts to shorten the preoperative waiting time after admission by establishing a green channel group for fractures, speeding up the inspection progress and strengthening multidisciplinary cooperation. Surgery should be performed as soon as possible after the patient's general condition stabilizes. Attention should also be paid to the time from the injury to admission. Primary hospitals should expedite the referral of patients if they are unable to provide appropriate medical treatment owing to insufficient resources. Furthermore, the time from injury to admission should be noted. Primary hospitals should speed up the referral of patients if they are unable to offer appropriate medical treatment due to insufficient resources.

Collectively, both ASA grades (III and IV) and elevated CRP levels were risk factors in our study. There are few reports on whether ASA classification is significantly correlated with DVT formation. ASA grade is a routine assessment of the physical condition, anesthesia, and surgical tolerance of inpatients. Previous studies^19^ have reported the indispensable role of ASA classification in predicting mortality and complication risk after hip fractures. Zhang et al.^20^ conducted a prospective study of 160 elderly patients with distal femoral fractures complicated by DVT and determined that ASA grade was an independent risk factor for DVT (OR = 2.753, *p* = 0.026). A national survey of DVT and PE after colorectal surgery in the United States^21^ showed that ASA grade (>II) was a risk factor for DVT after colorectal surgery. There are still few reports on whether ASA classification is significantly correlated with DVT formation. The reason for this condition is believed to be that the ASA classification is mainly evaluated by the comprehensive medical history and subjective judgment of the doctor, which is not an objective index.

CRP is a non‐specific inflammatory protein marker, which rises sharply in plasma when the body is affected by infection or tissue injury and plays an important protective role in the formation of innate immunity in the body.^22^ The American Heart Association recommends that elevated CRP levels may be a predictor of coronary heart disease and unsuspected serious non‐vascular disease in adults.^23^ Zhang et al.^20^ found that patients with hip fractures with CRP levels >11 mg/L had a higher probability of preoperative DVT. Bakirci et al.^24^ showed that the occurrence of DVT before and after an ankle fracture was closely related to an increase in CRP. In addition, CRP has been shown to be associated with D‐dimer because D‐dimer and other fibrin degradation products can upregulate the synthesis of interleukin‐6, thereby promoting CRP synthesis.^25^

For some reason, the upper limit of D‐dimer test results in our hospital was 10 mg/L, and we converted it into classified data (D‐dimer<5 mg/L group and D‐dimer ≥5 mg/L group) for further study. D‐dimer is a fibrinolytic enzyme‐soluble, cross‐linked fibrin‐specific degradation product. An increase in D‐dimer level indicates the presence of hypercoagulability and secondary hyperfibrinolysis in the body. It can be used as an auxiliary diagnostic index for thrombotic diseases and has remarkable significance in the diagnosis of CMVT.^26^ Individual D‐dimer has a high sensitivity for predicting thrombosis, but its specificity is low, and it is often used as a reference index clinically.^27^ D‐dimer level is often used as an exclusion index. D‐dimer <0.5 mg/L can exclude the presence of thrombosis with 95% accuracy. Studies have reported that the optimal D‐dimer cut‐off value for predicting DVT in hip fractures should be 1.0 mg/L, which is about twice the standard upper limit (0.5 mg/L).^21^ The risk of preoperative DVT in patients with hip fracture with D‐dimer >1.0 mg / L increased by 3.5 times. Combining D‐dimer levels with other indicators (such as age or CRP) can further improve the accuracy of DVT prediction.^28^ A recent study indicated that the best‐supported cutoff D‐dimer value was 1.59 mg/L based on ROC analysis.^29^

### Strengths and Limitations

As we known, our study was first to investigate the prevalence and risk factors for CMVT in elderly patients with hip fractures before surgery. We believed that our findings could help facilitate the peri‐operative management of patients.

However, it is necessary to highlight the following deficiencies of the present study: (1) This study was based on data from the electronic medical record system. When collecting data, patients without complete data were excluded (most lacked color Doppler ultrasound results). Based on clinical experience, the absence of examinations for these patients was mostly due to a lack of suspicious symptoms or economic reasons. Incomplete data may have affected our analysis of disease incidence and risk factors to some extent. (2) More patients with coronary heart disease or cerebrovascular disease and the need for routine use of aspirin and other drugs. Such inpatients routinely stop taking relevant drugs after admission until surgery; therefore, this study did not exclude such patients.^30^ (3) This study was conducted at a single institution. The sample size was relatively small. As the number of patients increases, the results of future risk factors for CMVT may differ from our results. Therefore, multi‐center, large‐sample prospective clinical studies are required in the future.

### Conclusion

Sex, time from injury to admission, ASA classification, CRP level, and D‐dimer level were independent preoperative predictors of CMVT in elderly patients with hip fractures. A prediction model based on these five factors showed good efficacy in the prediction of CMVT risk. This finding will help orthopaedic surgeons take preventive measures to avoid the occurrence and deterioration of CMVT in patients with these risk factors.

## Author Contributions

Sheng Pan, Shen Zhou, and Xie‐yi‐dai Ruze contributed equally to this work. Sheng Pan, Yang‐yu‐fan Wang, and Xin Zheng designed the study and drafted the manuscript. Shen Zhou, Da‐lin Peng, and Zi‐wen Yan performed the data collection and the statistical analysis. Xie‐yi‐dai Ruze, Wang‐yiJin, Zi‐wen Yan, and Kai‐jin Guo collected the information of patient samples. All authors read and approved the final manuscript.

## Conflict of Interest Statement

The author(s) declared no potential conflicts of interest with respect to the research, authorship, and/or publication of this article.

## Consent for Publication

All the authors in this study agreed to be involved and have agreed upon the submission and publication of this manuscript.

## Ethics Statement

This study was approved by the Clinical Trial Ethics Committee of Affiliated Hospital of Xuzhou Medical University (XYFY2021‐KL127‐01). All of the patients signed informed consent before surgery.
